# Vein of Galen Malformation—Experience of the Last 13 Years in a Reference Center from South-Eastern Europe

**DOI:** 10.3390/life15101536

**Published:** 2025-09-30

**Authors:** Ana Mihaela Bizubac, Maria Alexandra Fleaca, Mariana Carmen Herișeanu, Carmina Nedelcu, Alexandra Bratu, Veronica Marcu, Cristina Filip, Cătălin Cîrstoveanu

**Affiliations:** 1 Faculty of Medicine, Carol Davila University of Medicine and Pharmacy, 050474 Bucharest, Romania; ana-mihaela.bizubac@umfcd.ro (A.M.B.); maria-alexandra.fleaca@rez.umfcd.ro (M.A.F.); mariana-carmen.iliescu@drd.umfcd.ro (M.C.H.); carmina.georgescu@drd.umfcd.ro (C.N.); catalin.cirstoveanu@umfcd.ro (C.C.); 2 Neonatal Intensive Care Unit, “Marie-Sklodowska Curie” Children’s Emergency Hospital, 20 Constantin Brâncoveanu Street, District 4, 041451 Bucharest, Romania; alexbratu97@yahoo.com; 3 Department of Radiology, “Marie-Sklodowska Curie” Children’s Emergency Hospital, 20 Constantin Brâncoveanu Street, District 4, 041451 Bucharest, Romania; veronicamarcu@yahoo.com; 4 Department of Cardiology, “Marie-Sklodowska Curie” Children’s Emergency Hospital, 20 Constantin Brâncoveanu Street, District 4, 041451 Bucharest, Romania

**Keywords:** the vein of Galen, neonatal pulmonary hypertension, Bicêtre neonatal score, neonatal intensive care unit

## Abstract

The vein of Galen malformations (VoGMs) is mainly correlated with the retention of an embryonic pattern of vascularity, inducer of vein of Galen dilation, and formation of arteriovenous communications that give rise to the risk of systemic shunting, causing cardiac dysfunction, vascular steal, and venous hypertension. This is a rare cerebral vascular malformation in the newborn, accounting for 1% of all cerebral arteriovenous malformations and occurring in approximately 1 in 25,000–50,000 live births. We review nine cases of newborns diagnosed with vein of Galen malformations (VoGMs) to assess whether this pathology demonstrates a marked improvement over the past 13 years in diagnostic accuracy, treatment approaches, and patient survival rates within our clinic. Medical treatment was focused on providing inotropic support and tightly controlled peripheral and pulmonary vasodilation with the aim of overriding the effects of high output heart failure. Most of the patients underwent liver failure and flow-mediated pulmonary hypertension, while half of the newborns expressed anomalies of the nervous system due to impaired cerebral hemodynamics. Given the unavailability of endovascular treatment in our unit, which predisposes the newborns to a higher vital risk, we recognize the importance of delivering tailored intensive care aimed at maintaining cardiorespiratory and hemodynamic stability until a curative intervention can be performed in a specialized center.

## 1. Introduction

The aneurysmal variants of the vein of Galen represent rare choroidal malformations, accounting for less than 1% of all fetal vascular abnormalities, that develop at an early embryonic age, between the 6th and 11th week of gestation, and are mainly correlated with arteriovenous communications between the arterial network and the forerunner of the vein of Galen, the median prosencephalic vein of Markowski [1,2,3].

The retention of the embryonic pattern of vascularity causes dilation of the vein of Galen and formation of arteriovenous communications that give rise to systemic shunting, inducing alteration of the cardiac function, vascular steal into cerebral arteries, and venous hypertension [3,4]. Alternative venous drainage pathways typically develop to accommodate the markedly increased blood flow associated with the shunt, and the adequacy of these alternative pathways remains one of main determinants of long-term prognosis [5,6].

There are several systems for classifying malformations of the Galen vein (VoGMs), including that of Lasjaunias [7], which divides the malformations into the choroidal and the mural subtypes, or that of Yasargil [8], which defines four categories depending on the purely cisternal or multifistular appearance of the malformation with different drainage points into the vein of Galen.

During intrauterine life, the low-resistance placental circulation offset the blood flow through the aneurysm and keeps pressure variations under control [5,9]. The sudden increase in systemic vascular resistance at the time of delivery and the exclusion of low-resistance placental circulation result in higher diversion of flow throughout the malformation; therefore, vascular steal usually reveals itself only after the transition circulation has completed [8,9].

The development of cardiac failure is related to the magnitude of the arteriovenous shunt [4,10]. Correspondingly, evidence of progressive cardiac dysfunction in utero is indicative of future decompensation during extrauterine life in the context of a high-flow lesion, most likely to become unresponsive to therapy after birth [9,11].

### 1.1. Cardiopulmonary and Intracerebral Hemodynamics

Partial blood flow deviation to aneurysm causes a compensatory increase in the cardiac output in order to maintain systemic perfusion and subsequent enhanced pulmonary flow to improve the preload. This leads to pulmonary hypertension [12,13,14], which can be stratified into three cardiopulmonary phenotypes of VoGM: (1). flow-mediated pulmonary hypertension, (2). resistance-mediated pulmonary hypertension, and (3). isolated right ventricular dysfunction.

Flow-mediated pulmonary hypertension is generated by increased blood flow through the low resistance vessels of the VoGM that results in increased return via the superior vena cava and large volume load to the right ventricle [14], deriving into pulmonary hyperflow. Alongside magnified pulmonary return to the left atrium, it results in high cardiac output that will be further diverted to the vacuum of low-resistance vessels in the arteriovenous malformation. The final outcome is flow reversal in the proximal and distal arch, that becomes detrimental to systemic perfusion, leads to minimal peripheral oxygenation, lactic acidosis (pseudo aortic coarctation pathophysiology), and therefore dependency on the patency of ductus arteriosus.

Resistance-mediated pulmonary hypertension presents with similar augmented right cavities due to increased superior vena cava return out of the VoGM malformation. However, in this phenotype the pulmonary flow is restricted due to the reagent’s increased resistance in the lung vessels (pulmonary arterial hypertension flare pathophysiology); thus, the result is decreased preload of the left atrium and consequent insufficient left ventricle output. Right-to-left shunting also occur at the level of ductus arteriosus because of the suprasystemic rise in pulmonary arterial pressure [15]. This right-to-left type of shunt can significantly reduce diastolic pressure within the aorta, causing severe hypoxemia, reduced coronary flow, and therefore myocardial ischemia [8,11,15].

In neonates, the cavernous sinus is poorly developed and excluded from the venous drainage as it matures later and sylvian veins connect to it several months after birth; consequently, the entire venous drainage of the brain is temporarily supported by the central venous sinuses [2]. Arteriovenous malformations of the vein of Galen are usually associated with abnormal embryogenesis of these venous sinuses or secondary venous stenosis/occlusion, thereby influencing poor venous drainage [2,6,10].

As the arachnoid granulations have not yet fully matured, most of the ventricular cerebrospinal fluid of neonates and infants freely moves into the ependymal space of the ventricular wall and drains through the medullary veins [16]. In patients with VoGMs, in the context of restricted venous drainage, the presence of high-flow arteriovenous shunting leads to CSF accumulation and subsequent hydrocephalus, cerebral edema, and chronic hypoxia [17], conditions that become responsible for progressive cerebral parenchymal atrophy [18].

### 1.2. Diagnostic Algorithm and Therapeutic Approach

The differential diagnosis of VoGM includes arachnoid cysts, choroid plexus cysts, pineal tumors, choroid papilloma, cavum vergae (posterior extension of the cavum septum pellucidum), hemangiomas, and pial arteriovenous malformations [6,19].

In the first place, diagnosis is made by color Doppler ultrasound as the aneurysm of Galen’s vein is the only lesion from the above mentioned that displays blood flow within [20]. Cranial Doppler ultrasound also proved to be a reliable prognostic technique, used to measure certain indices, such as the maximal systolic velocity, the end-diastolic velocity, or the resistance index [21], but also to record flow reversal profiles in the superior sagittal sinus or veins [22]. Correlation with echocardiographic parameters for severity prediction (ratio of antegrade/retrograde flow in the aortic arch, superior vena cava augmented flow, dilated head and neck vessels off the aortic arch, indirect signs of suprasystemic pulmonary arterial pressure, dilated +/− dysfunctional right heart, reversal of flow through PDA) is mandatory in order to predict which neonates will escalate to severe heart failure, therefore imposing faster intervention for embolization [14].

Magnetic resonance (MR) imaging can assist in the early prenatal detection of VoGM or postnatal therapeutic management [23,24]. Computed tomography (CT) scanning can also help identify secondary findings (ventricular enlargement, brain atrophy, etc.) while CT angiography can provide an accurate reconstruction of the feeding and emerging vessels [19]. MR angiography has proven to be invaluable for prior treatment assessment of these lesions and post-interventional follow-up of incompletely removed malformations, remaining the gold standard for diagnosis [1,19].

Timely diagnosis of the vein of Galen malformation, particularly during the pre/perinatal period, and endovascular treatment in the early neonatal period is crucial in preventing heart failure and resulting morbimortality [13,24]. Once the diagnosis has been confirmed, interventions can be categorized as medical/non-surgical and procedural, including endovascular treatment or surgical resection [15,25]. Before endovascular techniques, mortality was reported up to 80–100% with neurosurgery only. The management of the aneurysmal variants of the vein of Galen poses a great challenge, especially if the cardiac function is compromised at the time of diagnosis [14].

Lasjaunias and co-workers, who have the largest experience in the management of these lesions, discussed therapeutic decisions based on their clinical expression in the neonatal period. They described a 21-point scale, later adapted to Bicêtre neonatal evaluation score (see attached Table S1 in manuscript—Supplementary) based on cardiac, cerebral, respiratory, hepatic, and renal function [4,7]. A score of < 8 usually indicates a poor prognosis and does not warrant an emergency intervention, but rather a palliative approach. A score of 8–12 is an indication for emergency endovascular management. A score > 12 indicates a well-preserved neonate and attempts are made to delay the endovascular procedure by supportive medication, especially in the case of premature babies [14].

Medical management should aim to counteract heart failure and postpone the endovascular intervention [14,15], which is considered to be the optimal therapeutic strategy, in the light of achieving complete occlusion; however, it should be performed after stabilization of the neonate and preferably after the 5th or 6th month of life, excluding conditions such as failure to thrive, unstable cardiac failure or progressive macrocrania, which are indications to advance embolization [4,26,27]. But nevertheless, about 30% of newborns are not candidates for early embolization for the following reasons: severe brain injury on postnatal MRI and concerns for cardiogenic shock or multiple organ failure despite optimization of medical treatment [14,27,28]. In addition to this, the outcome of vein of Galen malformation complicated by severe cardiac failure remains poor and even early embolization seems to be beneficial only in babies without suprasystemic pulmonary hypertension [11].

Efficient embolization often requires multiple consecutive procedures as complete occlusion may not be achieved in the first place [25,27,29]. A step-by-step intervention may also be justified to prevent abrupt increase in afterload and expected acute left ventricular systolic dysfunction.

The sudden shift in intracranial circulatory dynamics after embolization can also result in cerebral hyperperfusion, congestion, venous infarctisation, intracranial hemorrhage, and cerebral sinus thrombosis, leading to postoperative complications such as recurrent seizures, congestive heart failure or aggravation of hydrocephalus [29]. Choroidal VoGMs generally require a longer and more unpredictable recovery period than mural VoGMs [18].

Gamma knife radiotherapy has recently been used as a second-line therapy to reduce VoGM size and feeders after ineffective embolization, but in elder children [26,30]. However, radiosurgery is not recommended as a first-line treatment, especially in neonates or infants, since it requires time for inducing endothelial cell proliferation to support luminal closure and to provide hemodynamic relief [30,31].

Postoperative management should include vigorous respiratory and heart support, daily ultrasound monitoring of the hemodynamic status [20] through measurement of the cardiac output (ejection fraction > 45%), tissue Dopplers, diastolic markers, LV strain, flow patterns, close invasive monitoring of blood pressure, and assessment of cardiopulmonary symptoms and neurological deficits [18]. Modulating increased LV afterload and upregulation of the renin–angiotensin activating system by Milrinone intravenous administration alongside synergic inotrope and vasopressor agents after embolization can improve outcome in neonates with secondary cardiac failure, although the risk of precipitating systemic hypoperfusion and renal failure remains valid [27,32]. Arteriography for postembolization evaluation should be performed typically 12 to 24 months after the procedure [5,26].

With advances in imaging technology, cardiac care, developments in the field of interventional neuroradiology, and the availability of better post-procedure intensive care, this once non-treatable malformation of Galen’s vein has become at least potentially or partially curable nowadays.

It has been recently hypothesized that in utero treatment may determine a better prognosis in the neonatal period. The first fetoscopy cerebrovascular embolization were reported last year by two teams from Boston Children’s Hospital [33] and Necker Enfants Malades University Hospital [34] that successfully treated under ultrasound guidance fetuses with documented dilation of the falcine sinus associating great likelihood of later neonatal decompensation. Progressive reduction in the caliber of the vein and total cardiac output was later documented, with no evidence of hemorrhage or infarction, and no necessity for cardiovascular support or recurrence of the embolization process. Therefore, the prospects may prove favorable given the possibility of minimally invasive compensation for the hemodynamic adverse impact of Galen’s vein malformations before birth.

## 2. Materials and Methods

We consulted 38 relevant references in the literature regarding VoGM via PubMed Central (published not earlier than 2010) and analyzed at the same time our practice experience and evolving treatment strategies over the last 13 years. Consequently, we present this retrospective series of nine eloquent cases for the Galen vein malformation from NICU of Marie-Sklodowska Curie Emergency Hospital for Children in order to illustrate how this pathology has known an upward evolutionary trend in terms of diagnosis, treatment, and survival rate in our unit. The inclusion criteria in our case series were any suspicion of arteriovenous malformation notified at the level of the quadrigeminal cistern during prenatal morphological screening or any dilated, pouch-shaped structure contiguous with the sagittal sinus, displaying turbulent Doppler signal during transfontanellar ultrasound screening at admission following echocardiographic findings relevant for cardiac decompensation with diastolic steal from the aorta or unexplained cause of persistent pulmonary hypertension, irrespective of gestational age or associated conditions.

Within our small study, we aimed to stratify the clinical presentation of our patients using the Bicêtre neonatal score and systematize our diagnostic and therapeutical means over time in order to refine both our own strategies and those from other resource-limited settings lacking curative intervention.

## 3. Results and Discussion

The patients included in our report were predominantly male (7/9) and full-term newborns (6/9, only 2 preterm), originating from partially or entirely assessed pregnancies (except for one whose gestational age and maternal–fetal infectious status were unknown), born by cesarean section from cranial presentation. Most of them did not need reanimation maneuvers right after birth (only two requested positive pressure ventilation and another one was oxygen-supplemented).

**Figure 1:**
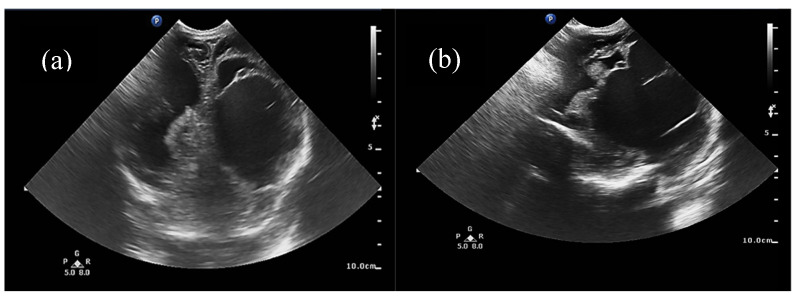
(**a**) Two-dimensional ultrasound of the VoGM via anterior fontanelle, (**b**) 2D ultrasound of the VoGM via the mastoid fontanelle. Voluminous, multilobulated, transonic mass of 6/3 cm, exerting a compressive effect on the left lateral ventricle, with slight displacement of the median structures.

**Figure 2:**
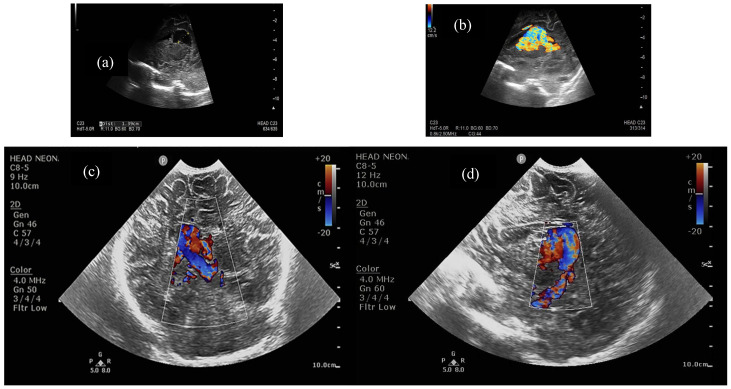
(**a**). Two-dimensional ultrasound close-up of the VoGM, (**b**). Color Doppler close-up of the VoGM, (**c**). Color Doppler in coronal transfontanellar section, (**d**). Color Doppler in parasagittal transfontanellar section. Median and right paramedian, supratentorial arteriovenous dilation of 10/28/20 mm, inferior to corpus callosum, with both arterial and venous supply, and suggestive appearance of an aneurysm of the Galen vein. Asymmetrical ventricular system, located on the midline. Focal lesions not visible at the level of the explorable cerebral parenchyma, except for small left parietal hematomas. Normally sized pericerebral spaces.

Half of the patients were diagnosed antenatally (by ultrasound or fetal MRI) and half of them postnatally, most frequently by cerebral ultrasound or after auscultation of a cardiac murmur followed by heart ultrasound displaying diastolic steal from the thoracic or descending aorta into the carotid territory towards cerebral circulation. The general aspect described was that of a saccular structure located at the level of the quadrigeminal tank with both arterial and venous Doppler signals, communicating with the large cerebral sinuses and having numerous arterial and venous collaterals (see Figure 1 and Figure 2).

**Figure 3:**
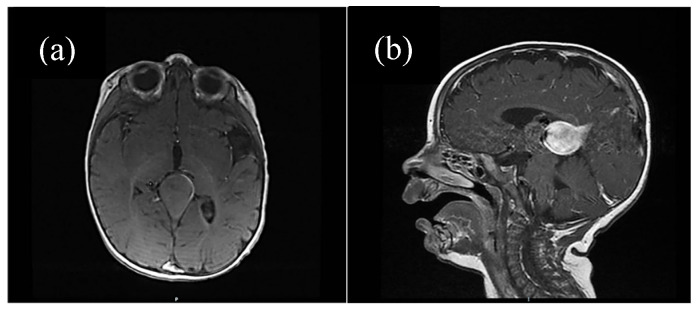
(**a**) Native cerebral MRI (transverse section), (**b**) contrast cerebral IRM (sagittal section). Vascular aneurysmal dilation at the level of the quadrigeminal tank, with no significant compressive effect, no thrombosis, no infra/supratentorial lesions. Asymmetrical ventricular system but normally positioned midline structures. Ependymal cyst located in the atrium of the LV. Asymmetry of the temporal lobes due to incomplete gyration.

**Figure 4:**
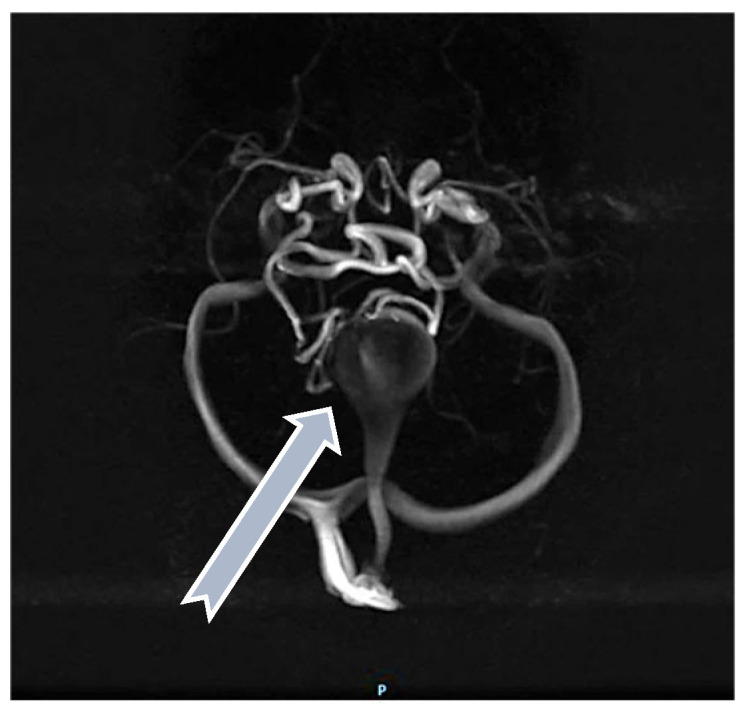
Angio-MRI sequence: Vascular aneurysmal dilation located at the level of the quadrigeminal tank (blue arrow), communicating with the large cerebral veins and the left middle cerebral artery, with collaterals emerging from the choroidal and posterior cerebral arteries—highly suggestive aspect of Galen vein aneurysm.

**Figure 5:**
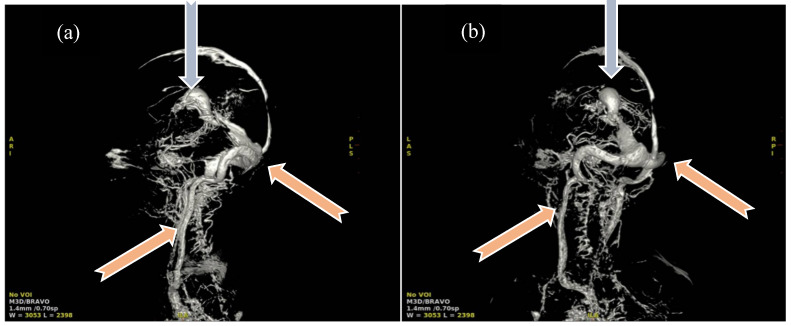
Three-dimensional reconstruction angio-MRI: (**a**) Sagittal view of the VoGM, (**b**) posteroanterior view in coronal plane. Aneurysmal median prosecephalic vein drained by the right sinus (blue arrows). Transverse/sigmoid sinuses and internal jugular veins of increased caliber (pink arrows). Multiple vascular pathways at the level of the basal nuclei connecting the anterior and posterior circulation. Carotid, vertebral arteries and basilar trunk exerting mass effect on the bulb.

**Figure 6:**
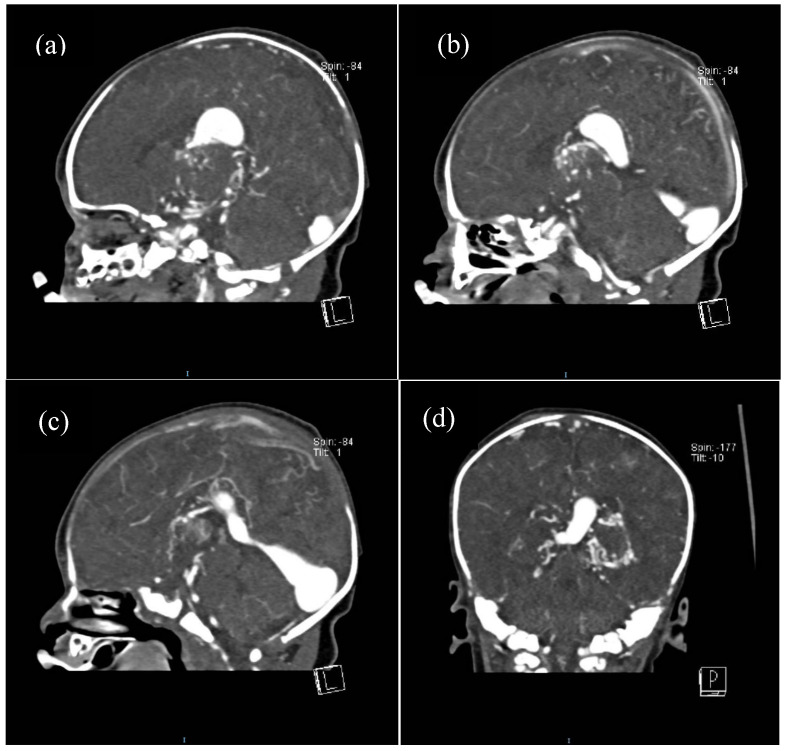
Contrast cerebral CT scan: (**a**–**c**). Progression of filling with contrast substance of the VoGM (sagittal view), (**d**). VoGM filled with contrast in coronal plane (posteroanterior view).

For some patients, the diagnosis was completed by brain MRI cerebral angio-CT (with iodinated radiocontrast agent at a concentration of 300 mg I/mL) methods for a better understanding of the Galen vein aneurysm morphology and its anatomical relations (see Figure 3, Figure 4, Figure 5 and Figure 6). Three of five patients who performed superior imaging techniques had large varieties of the Galen vein malformation, with variable degrees of compressive effect and secondary changes in the appearance of the midline and ventricular symmetry. Three patients presented with corpus callosum dysgenesis and three others with engorged arteries of the Willis polygon and enlarged pericerebral venous sinuses. None of the patients underwent an angiographic study of the arteriovenous malformation they had been diagnosed with in order to conceivably facilitate the perspectives of endovascular embolization in a center with expertise.

Most of the patients first received total parenteral nutrition, but managed to be eventually fed enterally via a nasogastric tube with maternal breast milk or formula. All patients maintained undisturbed intestinal transit and diuresis within normal limits, spontaneously or stimulated by Furosemide or/and Spironolactone (according to their electrolyte and fluid balance), not necessitating continuous renal replacement therapy in the context of cardiac decompensation.

Most of the patients underwent hepatic impairment from milder (cholestasis, hyperbilirubinemia with normal transaminase levels) to more severe forms (hepatic cytolysis, altered coagulation due to insufficient protein synthesis, hepatic/splenomegaly, ascites), and pulmonary hypertension within different cardiopulmonary phenotypes of the VoGM. All our patients needed respiratory support from a fairly early point in their evolution in order to reduce cardiac workload, seven of them undergoing chronic mechanical ventilation. A single patient (with a subtler form of VoGM in both size and cardiorespiratory implications) requested only non-invasive respiratory support for 48 h.

Half of the newborns expressed, in time, structural or functional anomalies of the nervous system (ventriculomegaly, diffuse hyperechogenicity of the infra/supratentorial white matter, attenuation of the cortical–subcortical differentiation, chronic ischemia of certain vascular territories, or cerebral atrophy) secondary to the arteriovenous malformation.

All patients had inserted central vascular access devices (5/10 both arterial and venous central catheters) for invasive measurement of arterial pressure and parenteral administration of nutrition or adjuvant medication (analgesics, diuretics, sedative hypnotics, inotropes, and vasopressors). Most newborns developed multicausal anemia, altered coagulation benchmarks due to acute hepatic failure arising from congestive cardiac failure, manifested a predilection for hemorrhage due to functional thrombopathy, thrombocytopenia of consumption, and altered synthesis of the coagulation factors, requesting multiple transfusions with red cell mass, fresh frozen plasma, platelets, albumin, and immunoglobulins.

Almost all patients had negative peripheral and catheter tip cultures, except for two babies who were tested positive for Acinetobacter and Staphylococcus aureus, respectively, in several collected biological products (skin samples and endotracheal aspirates). However, half of the patients had positive inflammatory markers (if not positive C reactive protein, then another specific marker such as procalcitonin or presepsin [35]) at some point during hospitalization in the NICU and needed a targeted antibiotic switch according to the antibiogram.

All the newborns with confirmed suspicion of intracerebral vascular malformation presented with unrelated, later diagnosed pathologies from the cardiovascular or nephrological sphere (see attached Table S4) that contributed more or less decisively to the clinical expression of the Galen vein malformation.

The most common cardiac pathologies documented among our patients were interatrial septum defect with or without interatrial septal aneurysm, sinus venosus atrial septal defect, patent ductus arteriosus with different types and degrees of shunting, and partial/total aberrant pulmonary venous drainage, which became the contributing factors to sooner or steeper decompensation. These findings were consistent with the literature [36,37]. A theory supporting these associations is that increased return via superior vena cava, inflicted by the aneurysmal Galen vein interposed at the level of cerebral circulation, may interfere in utero with the absorption of the right horn of the sinus venosus into the right atrium, which is assumed to be the developmental disturbance involved in the formation of sinus venosus atrial septal defect associated with anomalous pulmonary venous drainage.

None of the patients presented with aortic coarctation or hypoplasia although the literature often reports this association in the context of high cardiac output distributed largely to the brain, consistent with low-resistance circulation in the vein of Galen malformation, that subsequently leads to increased flow across the arterial duct and decreased anterograde flow to the isthmic aorta [36].

Medical treatment in our unit was focused on providing a balance between variable levels of inotropism, chronotropy, and peripheral vasoconstriction in certain situations (resistance-mediated pulmonary hypertension) of tightly controlled pulmonary vasodilation, with a specific intent of overriding high output heart failure. Inodilators (such as Milrinone and Dobutamine) were generally administered with moderation in selected cases marked by resistance-mediated pulmonary hypertension in order to reduce the negative effects of pulmonary overflow and to prevent vascular remodeling. Epinephrine, Norepinephrine, and Dopamine were the inotropic/vasopressor drugs of first election, and in isolated cases, Terlipresine and/or Levosimendan supplemented the therapeutic regimen as last resort. Vasoactive support was modulated to optimize end-organ perfusion, and evaluated through lactate levels and urinary output. Additionally, prostaglandin E1 infusion was indicated to keep the arterial duct open, while diuretics were used to reduce congestion.

Concerning respiratory support strategies, a careful balance between permissive ventilatory hyper/hypocapnia was kept by regulating vasoconstriction/vasodilation impact on the pulmonary vasculature. In flow-mediated pulmonary hypertension, we preserved higher pCO2s in order to increase vascular resistance in the lung by constricting mainly upstream vessels with the purpose of compensating pulmonary overflow; meanwhile, in resistance-driven pulmonary hypertension, lower pCO2s were ensured to induce controlled vasodilation in order to counteract the increased vascular resistance of the lungs. High-frequency oscillation ventilation was preferentially chosen when prematurity or risk of lung injury were associated, when conventional ventilation became inefficient and additional recruitment was needed, or when meconium aspiration syndrome was suspected. Switching to conventional ventilation was often considered if the patient presented with high airway resistance, severe sepsis, or became hemodynamically instable.

Inhalator nitric oxide was used for limited extents of time in the context of resistance-mediated pulmonary hypertension. A single patient’s hemodynamic status was worsened by the administration of iNO as flow-mediated pulmonary hypertension was actually the reason for refractory hypoxemia (the patient associated other cardiac abnormalities—large ventricular septal defect and partial anomalous pulmonary venous return).

We assessed our patients’ clinical status using the neonatal score of Bicêtre (see attached Table S1) at admission and sequentially during hospitalization, stratifying the hemodynamic impact of VoGM, and also evaluated paraclinically through daily transfontanellar and heart ultrasound.

Six patients diagnosed with a choroidal-type VoGM presented during hospitalization Bicêtre scores ranging from 4 to 7 (see attached Table S2). They escalated to severe heart failure refractory to medical treatment, which involved other forms of organ failure, or presented with neuroradiological risk factors, such as white matter lesions, hydrocephaly, and cerebral atrophy. For that reason, the indication for proceeding endovascular treatment in the neonatal period became an urgent necessity, but it was unable to be performed in our clinic.

Against this background, two-thirds of our patients died, except for three who were diagnosed with less extensive forms of mural type VoGM. One patient was a candidate for endovascular treatment and transfered during hospitalization. Two patients were discharged home in conditions of cardiorespiratory and hemodynamic stability with the possibility of postponing curative interventions until late infancy, when both were solved surgically. One developed mild–moderate neurologic impairment, but not necessarily correlated with the VoGM as he had a history of moderate perinatal hypoxia. The third survivor was not tracked in evolution under the circumstances of unsigned consent forms and ineffective communication with the parents. The surviving patients had a Bicêtre score between 16 and 20 (see attached Table S2). The deceased could not undergo minimally invasive embolisation or resection of the aneurysm quickly enough to prevent their abrupt degradation in the absence of a specialized center in our country, nor did they have a hemodynamic status that would make them transportable to centers with expertise.

## 4. Conclusions

Compared to the early days of VoGM diagnosis in Romania, when cases were often identified postnatally and significant systemic decompensation was already present, patients are now increasingly diagnosed antenatally and admitted to our NICU for supportive care until the optimal timing for curative intervention. However, the elevated morbimortality of this condition in our country enunciates a sad reality and raises the issue of prenatal underdiagnosis of this pathology with vital risk for newborns, even though progress has been made in terms of fetal screening and treatment. Even though the therapeutical lines applied in our clinic may be in accordance with the literature [14,25,26,27], no strict, unanimous, postnatal protocol, nor systematic monitoring during pregnancy is available.

In a setting lacking endovascular treatment opportunities, we focus our efforts on assessing the clinical impact of the VoGM at the time of diagnosis using the score of Bicêtre and intensive tailored management which can stabilize neonates with VoGMs until transfer is feasible. We want to create a model of nomogram for standardization of clinical states and predicting complications as it exists for other pathologies [38]. Therefore, we successfully implement targeted lung-protective invasive ventilation, recruitment techniques for preventing ventilation/perfusion mismatch, inhaled nitric oxide therapy, and non-invasive ventilation strategies in order to facilitate successful weaning and prevent bronchopulmonary dysplasia. We associate classic inotropes and vasopressors with inodilators for better ensuring end-organ perfusion. We prioritize volume restoration and counterweight of electrolyte imbalances, and during the recent years, we have become able to provide point-of-care ultrasound, NIRS and aEEG monitoring, efficient renal replacement therapies, and extracorporeal membrane oxygenation support before multiple organ failure set in. But even so, we are oftentimes moving rather towards symptomatic treatment, often belatedly to the moment when the major consequences of the malformation are already constituted, rather than focusing on prenatal diagnosis and diversion of the hemodynamic degrading pathway soon after birth. This is why we call out the need for more rigorous prenatal screening, including in rural, disadvantaged areas, where consanguinity is recurrent, and also for mediation of access to centers able to offer faster treatment options. In this direction, a referral protocol for eventual curative care should be standardized to include the clinical score of Bicêtre at the moment of diagnosis, paraclinical scores such as Vasoactive–Ventilation–Renal score, and one high-quality imaging technique mandatorily performed before requesting the transfer, in order to adequately assess the morphology of the malformation and frame its severity in the clinical picture.

Ultimately, we want to point out the scarcity of neurosurgical and neurointerventional expertise in our unit and the imperative need for collaboration with specialized centers that could provide treatment prospects or train our medical personnel, but also to acknowledge the progress we have made in terms of well-timed diagnosis and customized perioperative intensive care. Within the framework of such new advances in other endorsed units that are aimed at in utero endovascular embolization, we could at least challenge ourselves to attain the necessary aptitudes to treat this vascular anomaly after birth, but in due course, at a favorable distance in time from a potential irretrievable decompensation.

## Data Availability

The data presented in this study are available on request from the corresponding author.

## Supplementary Materials

The following supporting information can be downloaded at: https://www.mdpi.com/article/10.3390/life15101536/s1, Table S1: Bicêtre neonatal score for VoGM—classification by symptom group; Table S2: Bicêtre neonatal score for VoGM—applied to our patients; Table S3: Clinical and paraclinical features of our patients; Table S4: Associated pathologies with VoGM in our patients.

## References

[1] Raybaud C.A., Hald J.K., Strother C.M., Choux M., Jiddane M. (1987). Les anévrysmes de la veine de Galien. Etude angiographique et considérations morphogénétiques [Aneurysms of the vein of Galen. Angiographic study and morphogenetic considerations]. Neurochirurgie.

[2] Pearl M., Gregg L., Gandhi D. (2011). Cerebral venous development in relation to developmental venous anomalies and Vein of Galen aneurysmal malformations. Semin. Ultrasound CT MRI.

[3] Raybaud C.A., Strother C.M. (1986). Persisting abnormal embryonic vessels in intracranial arteriovenous malformations. Acta Radiol. Suppl..

[4] Garcia-Monaco R., De Victor D., Mann C., Hannedouche A., Terbrugge K., Lasjaunias P. (1991). Congestive cardiac manifestations from cerebrocranial arteriovenous shunts. Endovascular management in 30 children. Childs Nerv. Syst..

[5] Jones B.V., Ball W.S., Tomsick T.A., Millard J., Crone K.R. (2002). Vein of Galen aneurysmal malformation: Diagnosis and treatment of 13 children with extended clinical follow-up. AJNR Am. J. Neuroradiol..

[6] Aboian M.S., Daniels D.J., Rammos S.K., Pozzati E., Lanzino G. (2009). The putative role of the venous system in the genesis of vascular malformations. Neurosurg. Focus.

[7] Lasjaunias P. (1997). Vascular Diseases in Neonates, Infants and Children.

[8] Gupta A.K., Varma D.R. (2004). Vein of Galen malformations: Review. Neurol. India.

[9] Patton D.J., Fouron J.C. (1995). Cerebral arteriovenous malformation: Prenatal and postnatal central blood flow dynamics. Pediatr. Cardiol..

[10] Lasjaunias P., Ter Brugge K., Ibor L.L., Chiu M., Flodmark O., Chuang S., Goasguen J. (1987). The role of dural anomalies in vein of Galen aneurysms: Report of six cases and review of the literature. AJNR Am. J. Neuroradiol..

[11] Chevret L., Durand P., Alvarez H., Lambert V., Caeymax L., Rodesch G., Devictor D., Lasjaunias P. (2002). Severe cardiac failure in newborns with VGAM. Prognosis significance of hemodynamic parameters in neonates presenting with severe heart failure owing to vein of Galen arteriovenous malformation. Intensive Care Med..

[12] Devarajan A., Le C., Shigematsu T., Berenstein A., Fifi J.T., Morgenstern P.F. (2025). Obstructive hydrocephalus due to high-volume liquid embolic agent embolization for management of pediatric arteriovenous malformations: Illustrative case. Childs Nerv Syst..

[13] Patel N., Mills J.F., Cheung M.M., Loughnan P.M. (2007). Systemic haemodynamics in infants with vein of Galen malformation: Assessment and basis for therapy. J. Perinatol..

[14] Cory M.J., Durand P., Sillero R., Morin L., Savani R., Chalak L., Angelis D. (2023). Vein of Galen aneurysmal malformation: Rationalizing medical management of neonatal heart failure. Pediatr. Res..

[15] Frawley G.P., Dargaville P.A., Mitchell P.J., Tress B.M., Loughnan P. (2002). Clinical course and medical management of neonates with severe cardiac failure related to vein of Galen malformation. Arch. Dis. Child. Fetal Neonatal Ed..

[16] Zerah M., Garcia-Monaco R., Rodesch G., Terbrugge K., Tardieu M., de Victor D., Lasjaunias P. (1992). Hydrodynamics in vein of Galen malformations. Childs Nerv. Syst..

[17] Paramasivam S. (2021). Hydrocephalus in Vein of Galen Malformations. Neurol India.

[18] Hansen D., Kan P.T., Reddy G.D., Mohan A.C., Jea A., Lam S. (2016). Pediatric knowledge update: Approach to the management of vein of Galen aneurysmal malformations in neonates. Surg. Neurol. Int..

[19] Thomas J.A., Mitesh V.S. (2009). Chapter 16—Pediatric Central Nervous System Vascular Malformations. Stroke in Children and Young Adults.

[20] Vaksmann G., Decoulx E., Mauran P., Jardin M., Rey C., Dupuis C. (1989). Evaluation of vein of Galen arteriovenous malformation in newborns by two dimensional ultrasound, pulsed and colour Doppler method. Eur. J. Pediatr..

[21] Meila D., Lisseck K., Jacobs C., Lanfermann H., Brassel F., Feldkamp A. (2015). Cranial Doppler ultrasound in Vein of Galen malformation. Neuroradiology.

[22] Schwarz S., Nuñez F.B., Dürr N., Brassel F., Schlunz-Hendann M., Feldkamp A., Rosenbaum T., Felderhoff-Müser U., Schulz K., Dohna-Schwake C. (2023). Doppler Ultrasound Flow Reversal in the Superior Sagittal Sinus to Detect Cerebral Venous Congestion in Vein of Galen Malformation. AJNR Am. J. Neuroradiol..

[23] Amar B., Joshua A.C., Mary E.D., Helen F., Eduard G., Deborah K., Anthony O.O., Lawrence D.P., Boris T. (2018). Chapter 42—Vascular Cerebral Anomalies. Obstetric Imaging: Fetal Diagnosis and Care.

[24] Arko L., Lambrych M., Montaser A., Zurakowski D., Orbach D.B. (2020). Fetal and Neonatal MRI Predictors of Aggressive Early Clinical Course in Vein of Galen Malformation. AJNR Am. J. Neuroradiol..

[25] Khullar D., Andeejani A.M., Bulsara K.R. (2010). Evolution of treatment options for vein of Galen malformations. J. Neurosurg. Pediatr..

[26] Hosmann A., El-Garci A., Gatterbauer B., Bavinzski G., Knosp E., Gruber A. (2018). Multimodality Management of Vein of Galen Malformations-An Institutional Experience. World Neurosurg..

[27] Berenstein A., Fifi J.T., Niimi Y., Presti S., Ortiz R., Ghatan S., Rosenn B., Sorscher M., Molofsky W. (2012). Vein of Galen malformations in neonates: New management paradigms for improving outcomes. Neurosurgery.

[28] Melo-Guzmán G., Mallol-Valerio D., Soto-Barraza J., Granados-Hernández A., Taveras-González R. (2022). Arterial approach in vein of Galen aneurysmal malformation: Coils and EVOH. Interdiscip. Neurosurg..

[29] Pearl M., Gomez J., Gregg L., Gailloud P. (2010). Endovascular management of vein of Galen aneurysmal malformations. Influence of the normal venous drainage on the choice of a treatment strategy. Child’s Nerv. Syst..

[30] Brassel F., Schlunz-Hendann M., Scholz M., Lucaciu R., Fan C., Koch V., Grieb D., Brevis Nunez F., Schwarz S., Sommer C.M. (2023). Neurointerventional Treatment of Vein of Galen Malformation (VGM): A Structured Review with a Proposal for the Comparison of Outcome Quality. J. Vasc. Dis..

[31] Lucke-Wold B., Reddy R. (2022). Primer of Vein of Galen Malformation Management. J. Pediatr. Health Care Med..

[32] De Rosa G., De Carolis M.P., Tempera A., Pedicelli A., Rollo M., Luca E., De Luca D., Conti G., Piastra M., Morena T.C. (2019). Outcome of Neonates with Vein of Galen Malformation Presenting with Severe Heart Failure: A Case Series. Am. J. Perinatol..

[33] Orbach D.B., Wilkins-Haug L.E., Benson C.B., Tworetzky W., Rangwala S.D., Guseh S.H., Gately N.K., Stout J.N., Mizrahi-Arnaud A., See A.P. (2023). Transuterine Ultrasound-Guided Fetal Embolization of Vein of Galen Malformation, Eliminating Postnatal Pathophysiology. Stroke.

[34] Naggara O., Stirnemann J., Boulouis G., Orbach D.B., Grévent D., James S., Boddaert N., Kossorotoff M., Blauwblomme T., Ville Y. (2024). Prenatal treatment of a vein of Galen malformation by embolization and 1-year follow-up. Am. J. Obs. Gynecol..

[35] Stoicescu S.M., Mohora R., Luminos M., Merisescu M.M., Jugulete G., Nastase L. (2019). Presepsin-New Marker of Sepsis Romanian Neonatal Intensive Care Unit Experience. Rev. De Chim..

[36] McElhinney D.B., Halbach V.V., Silverman N.H., Dowd C.F., Hanley F.L. (1998). Congenital cardiac anomalies with vein of Galen malformations in infants. Arch. Dis. Child..

[37] Van Praagh S., Geva T., Lock J.E., Nido P.J., Vance M.S., Van Praagh R. (2003). Biatrial or left atrial drainage of the right superior vena cava: Anatomic, morphogenetic, and surgical considerations--report of three new cases and literature review. Pediatr. Cardiol..

[38] Liang L.X., Liang X., Zeng Y., Wang F., Yu X.K. (2025). Establishment and validation of a nomogram for predicting esophagogastric variceal bleeding in patients with liver cirrhosis. World J. Gastroenterol..

