# Supplementary material for: Vein of Galen Malformation—Experience of the Last 13 Years in a Reference Center from South-Eastern Europe
Source: Life (Basel). 2025 Sep 30;15(10):1536. doi: 10.3390/life15101536 (PMC12565294; doi:10.3390/life15101536)
Supplement: Supplementary file 1 [file life-15-01536-s001.zip › life-3808646-supplementary.pdf]

Table S1 ***Bicêtre neonatal score for* VoGM** – classification by symptom group

Points	Cardiac function	Cerebral function	Respiratory function	Hepatic function	Renal function
**5**	normal	normal	normal	–	–
**4**	overload, no medical treatment	subclinical, isolated EEG abnormalities	tachypnea, finishes baby bottle	–	–
**3**	heart failure, stable with medical treatment	non-convulsive, intermittent neurologic signs	tachypnea, does not finish baby bottle	no hepatomegaly, normal hepatic function	normal
**2**	heart failure, not stable with medical treatment	isolated convulsion	assisted ventilation, normal saturation $FiO_2 < 0,3$	hepatomegaly, normal hepatic function	transient oliguria/anuria
**1**	necessity for invasive mechanical ventilation	seizures	assisted ventilation, normal saturation $FiO_2 > 0,3$	moderate or transient hepatic failure	unstable diuresis with medical treatment
**0**	resistant to medical therapy	permanent neurological signs	assisted ventilation, repetable desaturations	abnormal coagulation, elevated enzymes	anuria

Table S2 ***Bicêtre neonatal score for*** **VoGM** – applied to our patients

Nr. crt.	Patient indicative	Cardiac function	Cerebral function	Respiratory function	Hepatic function	Renal function	***Total score***
1.	**Patient 1**<br>☐ male, at term	no cardiac failure	normal neuro examination for age	no HTP, no respiratory failure, no acidosis	normal transaminase levels, hyper-bilirubinemia	normal diuresis, normal creatinine and urea levels, normal renal ultrasound	**19**
Score for each vital function		+**5**	+**5**	+**3**	+**3**	+**3**	
2.	**Patient 2**<br>☐ male, at term	dilated right cavities, heart failure stable with medical treatment, elevated levels of CK, CK-MB and troponin T	normal neuro exam for age, *influenced* by analgesia and sedation	assisted ventilation, normal saturation FiO2 < 0,3 moderate to severe flow mediated HTP, no acidosis	temporary multiple etiology jaundice, no hepatomegaly, normal hepatic function	normal diuresis, normal creatinine and urea levels	**16**
Score for each vital function		+**3**	+**5**	+**2**	+**3**	+**3**	
3.	**Patient 3**<br>☐ male, unknown gestational age<br>*pregnancy without follow-up	heart failure resistant to medical therapy, severely dilated	subclinical, isolated EEG abnormalities, moderate	assisted ventilation, repetable desaturations, moderate to	abnormal transaminase levels, altered coagulation	unstable diuresis with medical treatment, no nitrogen	**5**

		right cavities, right to left shunt via PDA and PFO, diastolic retrograde flow, severe tricuspid insufficiency	perinatal asphyxia, corpus calosum dysgenesia	severe flow and resistance mediated HTP, mixed acidosis		retention, normal creatinine and albumin levels	
Score for each vital function		+**0**	+**4**	+**0**	+**0**	+**1**	
4.	**Patient 4**<br>☐ male, premature baby	severe congestive cardiac failure resistant to medical therapy	subclinical, isolated EEG abnormalities, present (ventriculo-megaly, cerebral atrophy)	assisted ventilation, repetable desaturations, moderate to severe resistance mediated HTP, (chronic) respiratory failure, mixed acidosis	hyper-bilirubinemia, hepatic cytolysis	transient diminuated urinary out, spontaneously recuperated, elevated creatinine, nitrogen retention	**7**
Score for each vital function		+**0**	+**4**	+**0**	+**1**	+**2**	
5.	**Patient 5**<br>☐ male, at term	global cardiomegaly with decreased contractility, resistant to medical therapy,	subclinical, isolated EEG abnormalities, left cerebral hypoplasia	assisted ventilation, repetable desaturations, unspecified HTP	cholestasis, conjugated hyper-bilirubinemia, hepatic cytolysis	unstable diuresis with medical treatment, elevated creatinine, normal urea	**6**

		elevated CK and CK-MB					
Score for each vital function		+**0**	+**4**	+**0**	+**1**	+**1**	
6.	**Patient 6**<br>☐ male, at term	severely dilated right cavities, congestive cardiac failure resistant to medical therapy, elevated CK, CK-MB, NT-proBNP	non-convulsive, intermittent neurologic signs (secondary cerebral lesions)	assisted ventilation, repetable desaturations, severe flow and resistance mediated HTP, metabolic acidosis	jaundice with conjugated hyper-bilirubinemia, hepatic cytolysis, hepatic failure	unstable diuresis with medical treatment, elevated creatinine, nitrogen retention	**5**
Score for each vital function		+**0**	+**3**	+**0**	+**1**	+**1**	
7.	**Patient 7**<br>☐ male, at term	no cardiac failure	normal neuro exam for age	no HTP, no respiratory failure, no acidosis	normal transaminase levels, no cholestasis or hepatic cytolysis	normal diuresis, no nitrogen retention, normal creatinine and albumin levels	**20**
Score for each vital function		+**5**	+**5**	+**4**	+**3**	+**3**	
8.	**Patient 8**<br>☐ female, premature baby	severe right ventricular hypertrophy, severe cardiac	non-convulsive, intermittent neurologic	assisted ventilation, repetable desaturations,	hepatic failure	unstable diuresis with medical treatment,	**4**

		failure resistant to medical therapy, paroxistic supraventricular tachycardia	signs (diffuse cerebral lesions)	severe resistance mediated HTP, pleural effusion, mixed acidosis		renal acute failure, hypo-albuminemia	
Score for each vital function		+**0**	+**3**	+**0**	+**0**	+**1**	
9.	**Patient 9**<br>☐ female, at term	right cavities dilated, congestive cardiac failure, rhythm disturbances, refractory hipoTA	normal neuro exam for age, *influenced* by analgesia and sedation	assisted ventilation, repetable desaturations, severe flow and resistance mediated HTP, pleural effusion, metabolic acidosis	cholestasis, hepatic cytolysis, hyperbili, hepatic failure, ascites	unstable diuresis with medical treatment, hypo-albuminemia	**6**
Score for each vital function		+**0**	+**5**	+**0**	+**0**	+**1**	

Table S3 ***Clinical and paraclinical features of our patients***

Aspects regarding the pathological context		Patients concerned	
Reanimation at birth		4/9	44,44%
Diagnosis	antenatally	4/9	44,44%
	postnatally	5/9	55,55%
Vascular catetherism	central arterial catether	4/9	44,44%
	central venous catether	8/9	88,88%
Presence of murmur	cardiac murmur	6/9	66,66%
	cerebral murmur	3/9	33,33%
Nutrition	total parenteral nutrition	3/9	33,33%
	enteral nutrition via NGT + PN	5/9	55,55%
	exclusive enteral nutrition	1/9	11,11%
Functional and structural imaging	thoracic and abdominal Rx	6/9	66,66%
	cerebral ultrasound	9/9	99,99%
	cardiac ultrasound	7/9	77,77%
	abdominal ultrasound	5/9	55,55%
	angio-CT	2/9	22,22%
	cerebral IRM	3/9	33,33%
	angiography procedure	0/9	0,00%
Diuretic medication	Furosemide as unique therapy	2/9	22,22%
	Furosemide, Spironolactone	4/9	44,44%
Sedation and analgesia	Phentanyl, Midazolam	5/9	55,55%
	Morphine	2/9	22,22%
Adjuvant medication	prostaglandin E2	3/9	33,33%
	Diazepam	1/9	11,11%
	Adenosine	1/9	11,11%
	Na bicarbonate	4/9	44,44%
	inotropic medication	7/9	77,77%
	vasoactive medication	5/9	55,55%
Blood products	red cell mass	5/9	55,55%
	fresh frozen plasma	7/9	77,77%
	plateletes	3/9	33,33%
	imunoglobulins	1/9	11,11%
	albumin	3/9	33,33%
Antibiotherapy	Cefazolin	1/9	11,11%
	Cefort	1/9	11,11%
	Aminoglycosides	2/9	22,22%
	Piperacillin, Tazobactam	6/9	66,66%
	Meronem, Vancomycin	6/9	66,66%
Inflammatory syndrome	negative CRP, negative PCT	4/9	44,44%
	negative CRP, positive PCT	1/9	11,11%
	positive CRP, positive PCT	4/9	44,44%
Acidosis	respiratory acidosis	1/9	11,11%
	metabolic acidosis	2/9	22,22%
	mixed acidosis	3/9	33,33%
Exitus		6/9	66,66%
Survival rate		3/6	33,33%

Table S4 ***Associated pathologies with* VoGM *in our patients***

Associated pathologies		Patients concerned
Atrial septal defect	single orifice	2/9
	double orifice	1/9
	with interatrial septum aneurysm	2/9
	without interatrial septum aneurysm	1/9
Patent foramen ovale	right to left shunt	2/9
	left to right shunt	0/9
Patent ductus arteriosus	right to left shunt	1/9
	left to right	1/9
	bidirectional	2/9
Anomalies of pulmonary artery trunk or branches	PA stenosis	1/9
	PA dilation	1/9
Anomalous pulmonary venous drainage		1/9
Ventricular septal defect		1/9
Valvular regurgitation	mitral regurgitation	2/9
	aortic regurgitation	1/9
Paroxistic supraventricular tachycardia		1/9
Infectious context	endotracheal colonisation	1/9
	maternal-foetal infection	2/9
	early onset neonatal sepsis	1/9
	late onset neonatal sepsis	0/9
Hemorrhages	superior digestive hemorrhage	1/9
	intraparenchymal hemorrhage	1/9
Cephalhematoma		1/9
Perinatal asphyxia		1/9
Hypovitaminosis D		1/9
